# Self-inflicted bilateral ocular injury with scissors in a psychiatric patient

**DOI:** 10.3205/oc000219

**Published:** 2023-05-23

**Authors:** Jigyasa Sahu, Ritu Arora, Shweta Vishwanath

**Affiliations:** 1 Guru Nanak Eye Centre, MAMC, New Delhi, India

**Keywords:** oedipus complex, deliberate self-harm, schizophrenia, self-mutilation, eye injury

## Abstract

A young male was brought to the ophthalmic emergency by his family with severe bleeding from both eyes after self-inflicted injury with scissors in a fit of mania. He had a history of schizophrenia and has been on irregular treatment for the past 10 years. Both eyes were severely damaged with avulsion of all extraocular muscles in one eye and a large corneoscleral laceration in the other. We report this unique case of oedipism or self-enucleation where a sharp instrument was used. Self-inflicted injury to the eyes is a rather uncommon phenomenon described in psychiatric patients. It can range from minor abrasions to severe ocular mutilation and loss of sight. The strict observation of these patients while admitted to the hospital and a multidisciplinary approach to ensure their future safety are imperative.

## Introduction

Self-inflicted ocular injuries are a rare phenomenon witnessed in ophthalmic clinics. Mostly associated with schizophrenia and psychosis, these cases are visually very disturbing to the ophthalmic surgeons themselves [1]. Traditionally referred to as oedipism, self-enucleation is the most common form of bilateral ocular mutilation reviewed in literature. In these cases the patients use their own fingers to “gouge” out their eyeballs due to a delusional belief [2]. Apart from this, few unique case reports of self-inflicted instrumental trauma using needles and razor to injure eyes have also been written [2]. Hence, the resultant damage in the majority of cases is permanent and beyond repair. 

Though most eye specialists will not encounter such an injury in their career, they should be well aware of the occurrence of such incidences. We describe one such unique case of self-inflicted severe bilateral ocular mutilation using scissors which has not been reported previously to the best of our knowledge. 

## Case description

**Figure 1:**
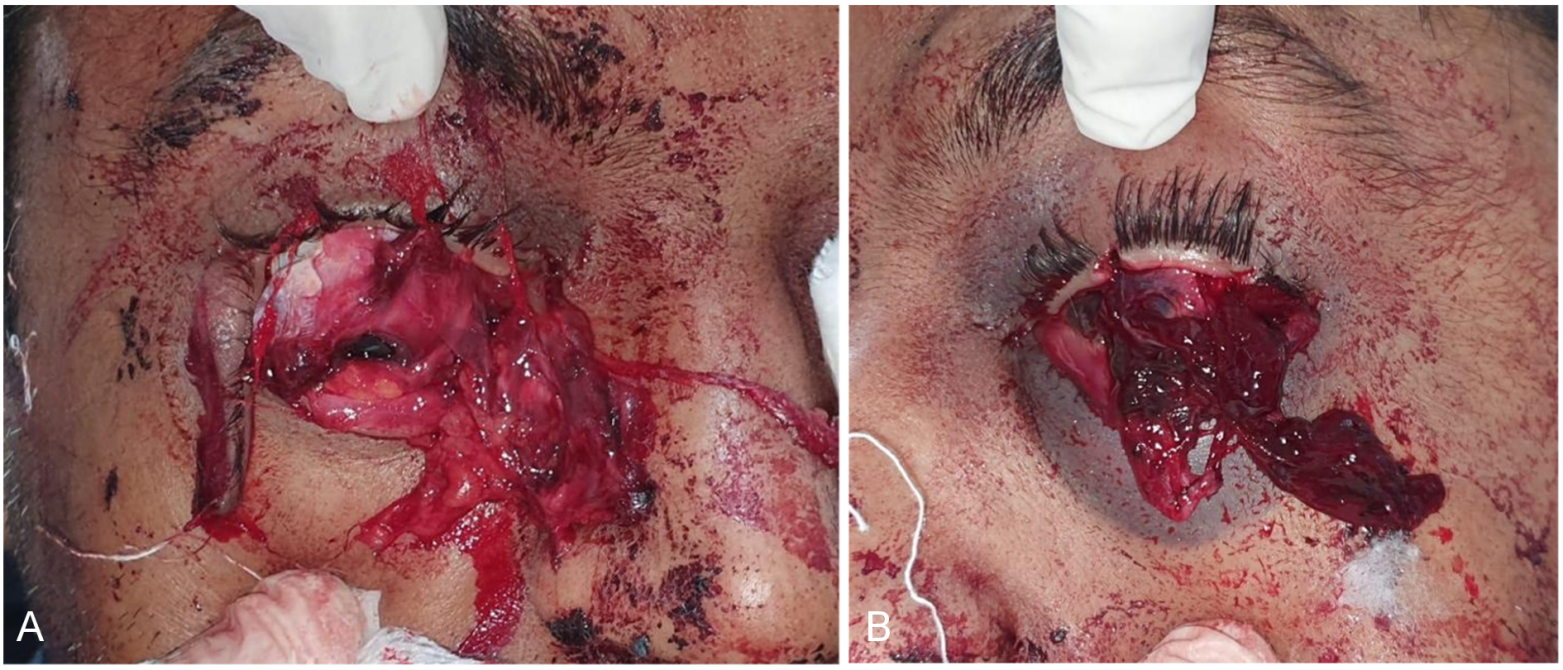
A: right eye showed avulsion and prolapse of all extraocular muscles sparing inferior rectus and oblique with near total full thickness detachment of lower eyelid; B: left eye showed large corneoscleral laceration with prolapse of vitreous and choroidal tissue

A 29-year-old male was brought to the emergency with an alleged history of self-inflicted injury with scissors to both his eyes by his family members. A collateral history from his family revealed that he had been on intermittent psychiatric treatment for manic-depressive disorder for the past 10 years. Due to side effects the patient had recently stopped taking treatment and had been on ayurvedic treatment irregularly. The family reported that he had isolated himself in his room for the past few days and told them that he was hearing external voices. On examination, there was extensive bleeding from both eyes and protrusion of ocular contents from the left globe. The right eye showed avulsion and prolapse of all extraocular muscles sparing inferior rectus and oblique with near total full thickness detachment of lower eyelid (Figure 1A (Fig. 1)). The cornea and anterior segment were intact and within normal limits. The left eye showed large corneoscleral laceration with prolapse of vitreous and choroidal tissue (Figure 1B (Fig. 1)). Visual acuity assessment revealed finger counting 1 metre in the right eye and no light perception in the left eye. At presentation the patient was in extreme pain and hence probably did not show any signs of agitation or psychotic behavior. 

**Figure 2:**
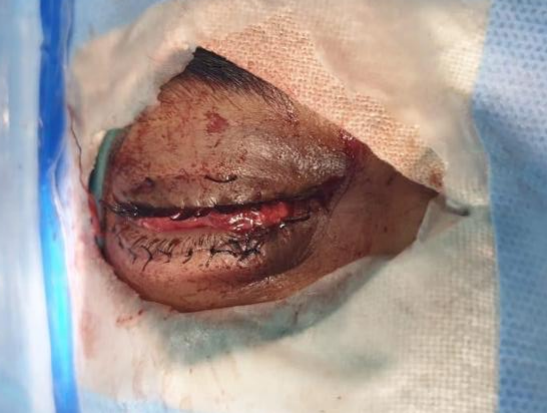
Right eye after primary lid repair and extraocular muscle repositioning

The patient was taken to the operating room and placed under general anaesthesia. Primary repair of the eyelid along with excision of prolapsed and necrosed extraocular muscle and orbital fatty tissue in the right eye was done in the first sitting as a sight saving procedure (Figure 2 (Fig. 2)). As there were constraints with GA time owing to the patient’s erratic psychiatric drug history, we decided to go with the potentially salvageable eye first to prevent risk of anterior segment ischemia. The more severely injured eye was PL negative and required evisceration, which was taken up as a second procedure. 

In the next sitting 2 days later, the left eye was explored which revealed a corneoscleral laceration in the inferior globe extending up to optic nerve insertion with prolapse of intraocular contents. The prolapsed ocular contents were removed, and a spherical implant was placed in the orbit after complete evisceration followed by closure of the overlying Tenon’s tissue and conjunctiva. In the same sitting, reattachment of the avulsed superior rectus and oblique of the right eye was also done. However, this was very difficult due to extensive distortion of muscular anatomy by avulsion and necrosis. The patient was then referred to the psychiatric ward and was put on immediate antipsychotic therapy. He was confirmed as a case of schizophrenia. His hospital stay was otherwise uneventful. The patient followed in our outpatient clinic for several months and was stable. The patient had a BCVA of 6/12 at one year post-op. However, the superior field of the patient was restricted with an elevation deficit owing to inferior rectus fibrosis.

## Discussion

The term “oedipism” was introduced by Bondel in 1906 to describe the phenomenon of ocular self-mutilation [1]. Self-inflicted eye injuries have been linked to psychotic depression and schizophrenia [2], epilepsy [3], encephalitis [4], LSD induced psychosis [5], Tourette syndrome [6], Lesch-Nyhan syndrome [7], and complicated diabetes mellitus [8]. Enucleation, lacerations, abrasions, induced traumatic cataracts, retinal detachments, and lens dislocations have been described. 

Maclean et al. [9] found in their review that most such cases were in mania at the time of the act. These patients who carry out self-harm often share a few common traits like acute psychotic symptoms, a belief that self-mutilation was essential to save themselves or prevent further harm, a lack of subsequent regret, religious dogma, delusion or hallucinations with religious or sexual themes, and substance abuse [10], [11]. Losing a father at an early age, having a dominant intrusive mother with whom they have unresolved oedipal conflicts, an alteration of body image, and sexual guilt were also found to be common in these patients. It is known that the notion of self-blinding for sinful thoughts also occurs in the Bible [12]: “Everyone who looks at a woman lustfully has already committed adultery with her in his heart. If your right eye causes you to sin, pluck it out and throw it away; it is better that you lose one of your members than that your whole body be thrown into hell.”

In our case, the young male was a case of manic depressive disorder from a young age. We did not find any positive family history. Rather intriguing was the use of scissors for performing the horrendous act, which has not been reported in literature earlier. It was quite puzzling to imagine whether the injury was done simultaneously to both eyes or sequentially. The lesser intensity of damage to one of the eyes made us believe that the act was sequential in both eyes. Soon after the act, the boy went into depression and did not interact much, hence more personal history could not be elicited. However, family members reported that he was fine and did not describe any kind of auditory hallucinations like before.

## Conclusion

Self-inflicted eye injury is a very rare ophthalmic and psychiatric emergency which warrants immediate attention and collaboration between the ophthalmologist and the psychiatrist because of the resultant severe ophthalmic and possibly lethal complications [13]. It is very important to insist on proper psychiatric treatment and cordial family relationships, especially in the case of such young patients.

## Notes

### Competing interests

The authors declare that they have no competing interests.
